# Supplementary material for: A Generic Method for Fast and Sensitive Detection of Adeno-Associated Viruses Using Modified AAV Receptor Recombinant Proteins
Source: Molecules. 2019 Nov 3;24(21):3973. doi: 10.3390/molecules24213973 (PMC6864843; doi:10.3390/molecules24213973)
Supplement: Supplementary file 1 [file molecules-24-03973-s001.pdf]

Supplementary table S1: Amino acid sequences of AAVR (PKD1-5) and SBP tag with the primers used for the construction of PET28a-AAVR-SBP-His plasmid

Amino acid sequence	
AAVR (PKD1-5)	MSAGESVQITLPKNEVQLNAYVLQEPPKGETYTYDWQLITHPRD YSGEMEGKHSQILKLSKLTPGLYEFKVIVEGQNAHGEGYVNVTV KPEPRKNRPPIAIVSPQFQEISLPTTSTVIDGSQSTDDDKIVQYHWE ELKGPLREEKISEDTAILKLSKLVPGNYTFSLTVVDSDGATNSTTA NLTVNKAVDYPPVANAGPNQVITLPQNSITLFGNQSTDDHGITSYE WSLSPSSKGKVVEMQGVRTPTLQLSAMQEGDYTYQLTVTDTIGQ QATAQVTVIVQPENNKPPQADAGPDKELTLPVDSTTLDGSKSSDD QKIISYLWEKTQGPDGVQLENANSSVATVTGLQVGTYVFTLTVKD ERNLQSQSSVNVIVKEEINKPPIAKITGNVVITLPTSTAELDGSKSS DDKGIVSYLWTRDEGSPAAGEVLNHSDHHPILFLSNLVEGTYTFH LKVTDAKGESDTDRTTVEVKPD
SBP tag	DEKTTGWRGGHVVEGLAGELEQLRARLEHHPQGQREP
Primer	
PKD1-5	Forward: caaatgggtcgggatatgtctgctggagagagtg
	Reverse: gccggtggtttttcatcatcaggtttcacctcc
SBP tag	Forward: tgtggaggtgaaacctgatgatgaaaaaccacc
	Reverse: tgctcgagtgcggccggttcacgctggc
